# Integration of substrate- and flow-derived stresses in endothelial cell mechanobiology

**DOI:** 10.1038/s42003-021-02285-w

**Published:** 2021-06-21

**Authors:** Claire A. Dessalles, Claire Leclech, Alessia Castagnino, Abdul I. Barakat

**Affiliations:** LadHyX, CNRS, Ecole polytechnique, Institut polytechnique de Paris, Palaiseau, France

**Keywords:** Biophysics, Cell biology

## Abstract

Endothelial cells (ECs) lining all blood vessels are subjected to large mechanical stresses that regulate their structure and function in health and disease. Here, we review EC responses to substrate-derived biophysical cues, namely topography, curvature, and stiffness, as well as to flow-derived stresses, notably shear stress, pressure, and tensile stresses. Because these mechanical cues in vivo are coupled and are exerted simultaneously on ECs, we also review the effects of multiple cues and describe burgeoning in vitro approaches for elucidating how ECs integrate and interpret various mechanical stimuli. We conclude by highlighting key open questions and upcoming challenges in the field of EC mechanobiology.

## Abstract (web summary)

Dessalles et al review the responses of vascular endothelial cells to mechanical forces exerted by both blood flow and physical contact with the basement membrane. Special attention is paid to how endothelial cells respond to multiple mechanical cues that are exerted simultaneously.

## Introduction

Research over the past two decades has established that mechanical forces are potent regulators of cellular structure and function in both health and disease. While all cells in our tissues experience physical forces, mechanical stimulation plays a particularly prominent role in the vascular system. By virtue of its strategic location at the interface between the bloodstream and the vascular wall, the endothelium is constantly subjected to a complex set of mechanical stresses that are often highly dynamic in nature. The ability of the endothelium to sense these biomechanical stimuli and to integrate information from different types of biophysical cues is essential for regulating vascular function.

**Fig. 1:**
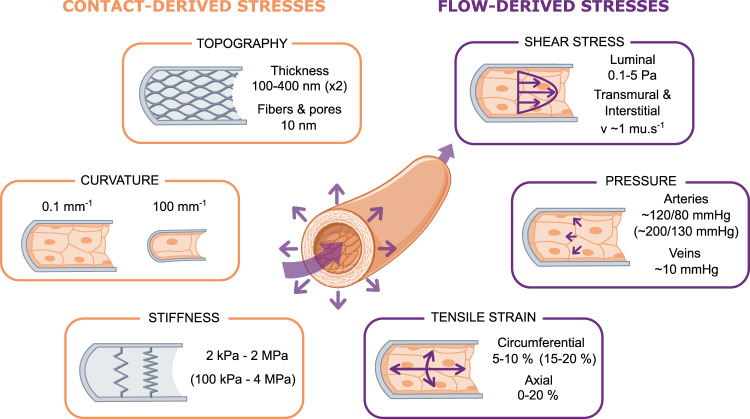
Summary of the biophysical forces present within the vasculature. Endothelial cells (ECs) within blood vessels are subjected to various mechanical cues, from the substrate (orange, left panels) or from the blood flow (purple, right panels). Physiological (or pathological in parentheses) ranges of values are provided for each type of cue.

The mechanical environment of the endothelium consists of a collection of intertwined stresses, which can be broadly divided into two categories (Fig. 1): contact stresses emanating from physical features of the underlying substrate and fluid-derived stresses due to blood flow. The contact stresses act on the endothelial cell (EC) basal surface and are principally due to substrate topography, curvature, and stiffness. The fluid-derived stresses consist of the shear stress on the EC apical surface due to the flow of viscous blood, the compressive blood pressure, and the circumferential and axial tensile stresses due respectively to the transmural pressure difference and tissue movement. All these stresses are dynamic in nature, albeit with considerably different time scales. While substrate physical properties at a given vascular location remain globally constant at short time scales, shear, pressure, and tensile forces are highly dynamic and vary cyclically due to blood flow pulsatility. In addition, the nature and magnitude of all these mechanical cues vary with location in the vasculature and across organs.

EC sensing and responsiveness to mechanical forces are critical for vascular homeostasis. How mechanical stimuli exerted on the EC surface are converted into intracellular biochemical responses has been reviewed elsewhere^1–3^ and will therefore not be detailed here. We can nevertheless mention that the various candidate mechanosensors in ECs can be categorized based on their cellular location. Apical mechanosensors include mechanosensitive ion channels^4–7^, primary cilia^8,9^, the glycocalyx^10^, GTP-binding proteins^9,11^, and caveole^12^. At cell–cell junctions, platelet endothelial adhesion molecule-1, vascular endothelial-cadherin, and vascular endothelial growth factor receptors have been shown to form an elaborate mechanosensory complex^13–15^. Integrins, the principal mechanosensors on the EC basal surface, provide a direct link between the actin cytoskeleton and the extracellular matrix (ECM)^16,17^ and are involved in the response to both substrate- and flow-derived cues^15^.

EC mechanobiology has been the subject of several excellent recent reviews, each with a specific focus: mechanotransduction events and governing mechanisms^2,3^, experimental platforms to mechanically stimulate ECs^3,18^, angiogenesis and vascular development^19^, mechanobiology of vascular diseases^20^, and physical/mechanical considerations^21,22^. Here we review EC responses to the different types of contact- and fluid-derived mechanical stresses present in the vasculature. We identify key features of each one of these biophysical cues and discuss how they affect EC morphology, intracellular organization, and overall function. We pay special attention to the interplay among these stresses and how ECs integrate multiple mechanical signals. Throughout the review, we focus on in vitro studies that have greatly advanced our understanding of EC mechanics and assess their physiological relevance and ability to mimic pathological conditions. We conclude by highlighting open questions and describing how recent technological advances promise to offer new avenues in the field of EC mechanobiology.

## Substrate-derived biophysical cues

In vivo, the vascular endothelium responds to various biophysical cues derived from the underlying vascular wall structure, most notably the topography of the vascular basement membrane (BM), the curvature of the tubular vessel wall, and the stiffness arising from the mechanical properties of the BM and adjacent cellular and connective layers (Fig. 1).

### Topography

Vascular ECs are anchored to a BM that is a few hundred nanometers thick and whose topography takes the form of intermingled fibers and pores^23,24^. The BM thus presents ECs with an isotropic topographical environment at the nanoscale, whereas at the microscale, the topography appears more anisotropic, formed by the organization of nanoscale structures and/or by tissue undulations^23,25^.

#### Influence of topography architecture: anisotropic vs. isotropic substrates

**Fig. 2:**
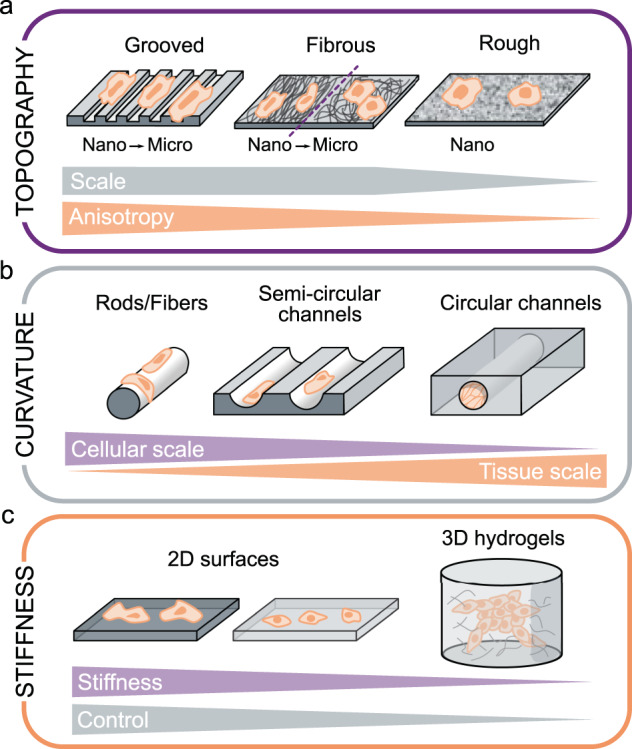
Substrate-derived cues experienced by endothelial cells and associated experimental model systems. **a** Substrate topography can be mimicked in vitro with grooved, fibrous, or rough surfaces. Each of these systems can provide a different level of anisotropy at different scales (nano- or micro-scale). **b** Substrate curvature can be mimicked in vitro with microscale rods or fibers, semicircular channels, or full circular channels. These systems provide curvature ranging from the cellular scale to the tissue (monolayer) scale. **c** Substrate stiffness can be tuned in either 2D surfaces (where increased cell spreading is observed on stiffer substrates) or 3D hydrogels (where smaller stiffness values can be attained but at the expense of more limited control of the extracellular environment).

Several types of engineered substrates have been developed to mimic the anisotropy found in some native extracellular environments. Despite being highly idealized configurations that differ considerably from physiological BM topographies, unidirectional grooved substrates consisting of parallel arrays of rectangular grooves and ridges have been widely used because their layout and dimensions can be precisely controlled, thereby providing well characterized biophysical cues to cells (Fig. 2a). Studies on these ridge/groove systems have provided crucial information about fundamental EC responses to anisotropic topographies. For instance, ECs have been shown to migrate, align, and elongate in the direction of grooved substrates^26–33^, reproducing EC morphologies encountered in vivo. Cell alignment and elongation are reflected intracellularly by the alignment of actin filaments^26,28,31–33^, microtubules^32^, and focal adhesions (FAs)^26,29,31^. Additionally, anisotropic grooved surfaces lead to stronger EC adhesion, more oriented migration, and greater migration speeds compared to isotropic surfaces (such as arrays of pillars or holes)^34,35^.

To assess the effect of the nanoscale BM roughness found in vivo, different types of rough substrates have been developed^36–38^ and have been shown to promote EC adhesion and growth relative to smooth surfaces^36^ (Fig. 2a). On these substrates, ECs are typically more elongated than on smooth flat surfaces but less so than on grooved substrates, and they migrate faster than on untreated surfaces^37^.

Fibrous networks are attractive model substrates as they resemble the physiological structure and composition of native BMs (Fig. 2a). Aligned (and thus anisotropic) fibers at both the nano- and microscale induce higher levels of cell and cytoskeletal alignment and elongation compared to random fibers^39–42^, as well as increased migration directionality^43,44^. From a more functional perspective, ECs on both aligned fibrous scaffolds^39,40^ and grooved substrates^30,40,45,46^ appear to adopt an anti-inflammatory and antiatherogenic phenotype.

#### Influence of topography dimensions: micro- vs. nanoscale cues

In vivo, the endothelial BM is a multi-scale topographic surface with both nanoscale fibers and microscale aligned or anisotropic topographies. Most studies suggest that decreasing groove width from the micron to the nanometer scale increases cell elongation, alignment, adhesion, proliferation, and FA size and stability^28,32,33^. In contrast, a similar decrease in groove depth decreases EC alignment and elongation^29,31^. On fibrous substrates, microscale fibers have been reported to induce both decreased^41^ and increased^42^ EC alignment and elongation relative to nanoscale fibers. This apparent contradiction may be explained by differences in pore sizes between the two studies, with cells capable of spreading over multiple fibers in the first study but not the second.

As cells usually encounter micro- and nano-cues simultaneously, combining both scales provides a more physiologically relevant environment. In a study that focused on this issue, nanofibers aligned in the same direction as microscale grooves were shown to have a synergistic effect, amplifying EC elongation and alignment, whereas an orthogonal orientation between nanofibers and microgrooves had an antagonistic effect^47^, demonstrating that ECs can integrate topographical cues of different scales.

#### Influence of EC type and cell density

ECs in vivo possess great phenotypic variability depending on the vascular bed and organs. Whether substrate topography induces different responses in different EC types remains unknown but is an important question given the differences in BM structure between arteries and veins^23,25^. A comparison of arterial, venous, and stem cell-derived ECs on grooves shows that while their morphological responses are similar, different phenotypic responses are observed: both primary cell lines preferentially adopt a venous phenotype, while grooves promote an arterial phenotype in stem cell-derived ECs^48^. The effect of groove size on the alignment and migration of ECs has also been shown to depend on EC type^34^, which may explain the discrepancies reported in the literature regarding the optimal topographic feature size for cell alignment.

An understudied aspect is EC response to topography in pathological settings. A recent study reveals that diabetic ECs are generally less responsive to topography than healthy ECs in terms of angiogenic capacity and monolayer integrity^49^. Similarly, ECs from older donors exhibit decreased cell speed but higher directionality along grooves compared to those from younger donors^50^.

ECs in vivo form continuous monolayers, while many of the previously mentioned in vitro studies focus on individual cells. Single ECs and EC monolayers exhibit broadly the same response to topography: they align and elongate along anisotropic substrates such as grooves or aligned fibrous scaffolds^30,32,35,39,40,45,51^. One study reported that EC alignment on grooved substrates decreased near confluence^31^, suggesting that ECs in monolayers are nevertheless less responsive to topographical cues than individual cells.

### Curvature

The impact of substrate curvature on cells has only recently been investigated and is now recognized as a critical cue regulating cell behavior^52^. In the vasculature, ECs encounter curvature at the subcellular, cellular, and tissue (monolayer) scales.

Subcellular curvature is due to the curvature of BM fibers and is typically studied using nano- to microscale wavy surfaces (sinusoidal grooves); however, only a few studies have so far used these types of substrates on ECs. In general, ECs align and elongate in the direction of sinusoidal grooves as they do on rectangular grooves, but they exhibit lower migration speeds^53^. EC alignment decreases progressively with increasing wavelength (i.e., decreasing curvature) until it disappears at a wavelength of ~50 µm^53–55^, a dimension comparable to EC size.

The curved vascular wall subjects ECs to cell- to monolayer-scale curvature. The diameter of human blood vessels ranges from ~5 µm in capillaries to more than 1 cm in large arteries, which translates to curvatures ranging from 0.1 to 100 mm^−1^, spanning four orders of magnitude. Starting with simple, non-perfused systems of semicircular channels, it was observed that ECs form confluent monolayers that conform to the curved substrate^56–58^ (Fig. 2b), orient along the longitudinal axis, and have fewer actin stress fibers compared to adjacent flat regions^56^. More recently, many “vessel-on-chip” systems have been developed where ECs are cultured in closed, perfused channels of physiological diameters (Fig. 2b)^59–61^, but the impact of substrate curvature on the cells in these systems has not yet been investigated.

The most informative studies on the impact of curvature on ECs arise from the use of microfibers over which ECs are cultured, mimicking the high curvature found in smaller vessels such as capillaries (Fig. 2b). While endothelial colony forming cells (ECFCs) were observed to orient in the direction of fibers with diameters of 5–11 µm^62^, human umbilical vein ECs (HUVECs) were reported to exhibit a circumferential orientation and ring-like actin organization on 5–20 µm PCL fibers^62,63^ but a longitudinal orientation on 14 µm glass rods^64^. In this last study, human brain microvascular ECs (HBMECs) retained a random orientation regardless of rod curvature. The authors hypothesized that this resistance to curvature might be an organ-specific behavior, allowing brain ECs to minimize the length of cell–cell junctions and hence to maintain very low permeability levels^64^. Interestingly, the combination of cell-scale curvature with nanoscale topography reveals competing effects: while nanotopography orientation appears to drive ECFC orientation, the orientation of the secreted ECM is driven by the cell-scale curvature^65^.

### Stiffness

An elastic material’s bulk deformability is characterized by its Young’s modulus, an intrinsic property defined as the ratio of stress to strain (SI units of Pa). Although the term “stiffness” is commonly used to indicate if a material is “soft” or “hard”, its technical definition is the ratio of an applied force to the elongation in the direction of the force (SI units of N/m). Stiffness thus depends on the material’s dimensions. In this review, the term “stiffness” corresponds to its common language usage. As such, the material stiffness values are actually Young’s moduli. It should be noted that in the literature, a clear distinction between stiffness and Young’s modulus is not always made.

Atomic force microscopy (AFM) measurements on excised adult BMs suggest that their elastic modulus is in the 1–4 MPa range^25^. A wide range of vessel wall stiffnesses has been reported, from ~10 kPa^66,67^ to 1.5 MPa^68^. It is important to emphasize that stiffnesses measured ex vivo in excised vessels differ from those in pressurized vessels in vivo^69,70^ due to the strain-stiffening behavior of extracellular matrices. Additionally, vessel wall stiffness changes with anatomical location and increases with age^71,72^ as well as with cardiovascular pathologies such as hypertension^73^ or atherosclerosis. The effective stiffness that ECs perceive in vivo is difficult to determine. Reports of the distance over which cells feel stiffness vary from a couple of microns to tens of microns^74,75^. By using soft gels on glass substrates, it has been shown that this distance depends both on the substrate’s Young’s modulus and the gel thickness^74–76^. Based on these studies, we can assume that large vessel ECs in vivo sense a combination of the stiffnesses of the BM and the underlying medial and adventitial layers, whereas ECs in small vessels might even sense the stiffness of adjacent tissues.

#### Influence of 2D substrate stiffness on EC morphological and functional responses

Varying substrate stiffness in vitro has a clear impact on EC morphology, with cells being more round and less spread on soft substrates (1–5 kPa) than on stiff substrates (20 kPa–2 MPa)^77–81^ (Fig. 2c). In terms of intracellular organization, ECs on softer substrates are associated with fewer FAs^79,80,82–84^ and actin stress fibers^77,80,82,85–88^. Furthermore, ECs on stiff substrates are more contractile and exhibit larger traction forces, both as single cells^81^ and in monolayers, as assessed by traction force microscopy^85,88,89^. The stresses within the monolayer are lower but are also more homogeneously distributed on soft substrates^80^.

Various studies have shown that monolayer and cell–cell junction integrity decrease with increasing substrate stiffness^79,80,85,89–91^, with important functional consequences for monolayer permeability and inflammation^89,92^. ECs in vivo can also encounter cell-scale heterogeneities in BM stiffness^66^. In vitro, gaps in EC monolayers were observed with increasing matrix stiffness heterogeneity, and tended to localize at matrix stiffness interfaces^84^, suggesting that stiffness gradients regulate EC structure and function.

#### Influence of EC type and cell density

Interestingly, although observed in both individual cells and monolayers, stiffness-related morphological and cytoskeletal modifications are considerably smaller in the case of monolayers^78,86^. This may reflect preferential force redistribution towards cell–cell junctions within monolayers. Although veins have softer walls than arteries^93^, it remains unknown if venous and arterial ECs respond differently to substrate stiffness. However, the switch to a venous phenotype observed for arterial ECs cultured on soft matrices (0.5 kPa) suggests that endothelial morphology and function are principally determined by the biomechanical properties of the ECM environment rather than intrinsic differences between arterial and venous ECs^83^.

#### Influence of 3D substrate stiffness on EC network formation

To better simulate the native extracellular environment, recent efforts have focused on developing 3D culture systems. Although the vascular endothelium can be viewed as an essentially 2D structure, 3D environments are particularly relevant in the context of angiogenesis, where new microvessels sprout from existing vessels in all directions (Fig. 2c). Studies using hydrogels of varying stiffnesses in the range of 0.1–10 kPa show enhanced EC vascular network formation in softer matrices^87,94–98^. Consequently, ECs migrate longer distances^99^ and are more spread in soft gels^94^, the opposite effect to that on 2D substrates. Importantly, tuning collagen gel stiffness by controlling the cross-linking rather than altering gel density and structure (as in the previous studies) yields the opposite effect: stiffer (500 Pa) collagen gels lead to increased spreading, number, length, and branching of angiogenic sprouts^90,100^. This observation highlights the importance and intrinsic difficulty of independently tuning the detailed features of the environmental cues, mainly stiffness and topography, in 3D culture systems. Using colloidal gels where stiffness and topography can be independently controlled, a recent study concluded that there is an optimal structure of tenuous strands and spacious voids for EC network formation, irrespective of matrix stiffness^97^. The magnitudes of the 3D substrate stiffnesses eliciting the aforementioned effects are approximately an order of magnitude lower than the 2D substrate stiffnesses, suggesting that matrix dimensionality modulates matrix stiffness effects. In any case, the apparent stiffness perceived by cells is hard to evaluate, as illustrated by the different morphologies of vascular networks seen in floating and constrained collagen gels that are otherwise identical^96^. In addition, while most of the different gels used in 3D studies possess purely elastic properties, the extracellular environment and its components in vivo exhibit viscoelastic and strain-stiffening behavior that are rarely reproduced in vitro, creating an additional layer of complexity for the physiological relevance of these models.

## Flow-derived mechanical cues

The endothelium experiences multiple mechanical stresses due to the flow of viscous blood. These include fluid dynamic shear (or frictional) stress, compressive pressure, and tensile (or hoop) stresses due to the transmural pressure difference (Fig. 1).

### Flow shear stress

Fluid shear stress is the tangential frictional force per unit area experienced by the endothelium as a result of blood flow. Blood is a complex non-Newtonian fluid composed principally of plasma and red blood cells. At sufficiently low shear rates (below ~100 s^−1^), blood exhibits shear-thinning behavior. Therefore, the non-Newtonian of blood strongly affects the shear stress on the EC surface within low shear regions^101–103^. In the microvasculature, blood can no longer be considered a homogeneous fluid but rather a suspension of deformable active particles^104–106^. Recent results indicate that in those vessels, the presence of blood cells can significantly alter the near-wall flow field, thereby impacting the shear stress on the EC surface^106^. Most of the in vitro studies discussed here document EC responses to physiological values of shear stress that are generated using more simple Newtonian fluids. Those investigations have revealed that shear stress regulates major EC functions including angiogenesis, vessel remodeling, and cell fate^19^. In vivo, the time-averaged wall shear stress is ~1 Pa in the aorta, ~5 Pa in small arterioles^1,107^, ~2 Pa in venules, and ~0.1 Pa in the vena cava^107–110^. Furthermore, within the same vessel, shear stress levels and profiles can vary significantly due to geometric features including vessel curvature and branching.

#### Steady flow: undisturbed vs. disturbed

**Fig. 3:**
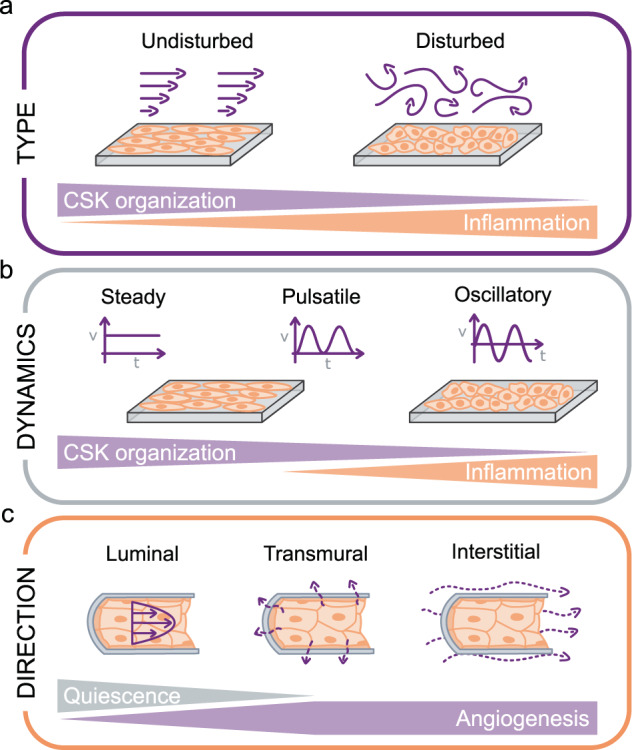
Features of flow-induced shear stress experienced by endothelial cells (ECs). **a** ECs experience either undisturbed or disturbed flow, which have different effects on cytoskeletal (CSK) organization and the inflammatory state of the cells. **b** ECs can experience steady, pulsatile, or oscillatory flow. The flow dynamics influence cytoskeletal organization and the inflammatory state of the cells. **c** ECs experience flow in the luminal direction (parallel to cells, on the apical side), transmural (across the endothelium, on cell–cell junctions), or interstitial (in the vessel wall or parenchymal tissue, on the basal side). The direction of the flow influences EC quiescence and/or angiogenesis.

Vascular flows can be broadly classified as either “undisturbed” or “disturbed”. Undisturbed flows are most typically uniaxial and laminar flows; whereas disturbed flows include turbulent flows and laminar flows with spatial shear stress gradients and/or secondary flows. In relatively straight vascular segments, flow streamlines are largely undisturbed and remain mostly parallel to the vascular wall^111^ (Fig. 3a). In contrast, flow in areas of vascular curvature, branching, and bifurcation becomes highly disturbed with regions of flow separation and recirculation (Fig. 3a). Interestingly, these zones of flow disturbance correlate with the localization of vascular diseases including atherosclerosis^112,113^, aortic valve calcification^114,115^, and inflammation and thrombosis in veins^115,116^.

In vivo, ECs in undisturbed flow zones are elongated and aligned in the direction of blood flow^117,118^. In contrast, ECs in disturbed flow areas are more cuboidal (round) and randomly oriented^119,120^. In vitro, steady flow systems are able to reproduce these observations, with undisturbed shear stress elongating ECs and aligning them parallel to the flow direction^4,121–125^ and disturbed shear stress leading to round EC shapes and random cellular orientation^121,126,127^. At the cytoskeletal level, undisturbed flow leads to prominent actin stress fibers that are aligned in the direction of flow^128–131^, whereas ECs subjected to disturbed flow exhibit shorter and randomly oriented actin filaments^4,121,123,130^. Shear-induced cytoskeletal reorganization is initiated within 1 h after flow onset^132^, even though complete cytoskeletal remodeling and cellular shape changes require significantly longer times^120^.

EC morphological and alignment responses to steady flow depend not only on the type of applied shear stress but also on shear stress magnitude. Although increasing shear stress level generally increases the extent of cell elongation and alignment^120,133,134^, there seems to be an optimal value of shear stress above which the response decreases^112^. Interestingly, several recent studies have challenged the consensus of EC alignment in the direction of steady flow, with reports of perpendicular alignment at both low shear (0.3 Pa)^135^ and high shear (>10 Pa)^136,137^. The alignment response may be dynamic, with a switch from perpendicular to parallel orientation after 72 h of flow^138^.

In addition to its effect on EC shape and cytoskeletal organization, steady unidirectional flow elicits higher EC migration speeds than disturbed flow^127,139^, leading to more efficient wound healing. This effect is dependent on shear stress magnitude^140^ and on the direction of cell migration, with ECs along the downstream wound edge migrating more slowly than ECs along the upstream edge^140^. ECs also appear to retain a level of shear stress memory, with pre-sheared ECs exhibiting accelerated wound closure^141,142^. Moreover, steady unidirectional flow promotes an anti-inflammatory and antithrombotic EC phenotype with reduced cell apoptosis and proliferation^143,144^ while disturbed flow has the opposite effect^126^.

Two additional and important features of disturbed flow that affect EC behavior are spatial shear stress gradients and secondary flows. Shear stress gradients inhibit EC alignment in the direction of the shear in vitro^138,145,146^. The alignment response can be restored, however, by supra physiological levels of shear stress^147^ whose value depends on the gradient magnitude^148^. EC alignment is also restored by switching from a positive to a negative gradient, underscoring the importance of gradient direction^149^. In a new type of “impinging flow device”, ECs were surprisingly observed to align perpendicular to high shear stress gradients^136^, emphasizing our incomplete comprehension of EC response to shear gradients.

Secondary flows associated with disturbed flow zones in regions of branching and curvature arise from streamline curvature. A key consequence of secondary flows for ECs is the generation of transverse shear stresses that act orthogonal to the axial shear stress due to the primary flow^150,151^. Interestingly, the localization of atherosclerotic plaques has recently been correlated with transverse flow rather than with disturbed axial flow^152^. The response of ECs to these secondary flows remains unclear with some reports of alignment loss in orbital shakers^153–155^. However, the transverse shear stresses in most of these systems are also accompanied by shear stress gradients and in some cases oscillatory shear forces^156^, thus complicating the isolation of individual effects and underscoring the need for the development of novel in vitro platforms that allow decoupling of these various phenomena. Recently, a unique system of spiral microvessels was developed in which physiological torsion and curvature enable the generation of physiological secondary flows^157^. This new generation of systems will certainly enhance our understanding of complex flow patterns and their influence on ECs.

#### Pulsatile flow: non-reversing vs. reversing vs. oscillatory

A key feature of blood flow in larger vessels is its pulsatility due to the rhythmic heartbeat. Within the microvasculature, this pulsatility is significantly dampened, and blood flow becomes quasi-steady. Thus, the shear stress profile on the EC surface depends on the vascular location. In undisturbed flow regions in medium and large vessels, the endothelium experiences non-reversing pulsatile flow, whereas in disturbed flow zones, the shear stress exhibits periodic directional reversal and oscillation.

ECs in vitro are able to discriminate among steady, non-reversing pulsatile, reversing pulsatile, and oscillatory (zero net) flow (Fig. 3b)^158–160^. For instance, while both steady and non-reversing pulsatile flow induce EC elongation and alignment in the flow direction^161,162^ as already mentioned, they do so with different dynamics^158^. The recruitment of apical stress fibers and the high migration persistence observed under steady flow disappear under non-reversing pulsatile flow^161^. Prolonged exposure to either reversing pulsatile flow or oscillatory flow fails to elicit EC elongation, orientation, and cytoskeletal remodeling^158,159,163^. Contrary to steady flow, oscillatory flow disrupts cell–cell junctions^125^ and elicits a pro-inflammatory and atherogenic EC phenotype^164–166^. These results underscore the need for understanding the links between the exact flow waveform and the resulting EC responses.

#### Luminal vs. transmural vs. interstitial flow

ECs in vivo are subjected to a combination of luminal, transmural, and interstitial flow (Fig. 3c). Luminal blood flow exerts a shear stress on the apical EC surface. The pressure difference across the vascular wall generates a transmural flow, particularly prominent in the microvasculature with typical flow velocities of ~1 µm/sec^167,168^. Transmural shear forces are exerted most directly on endothelial cell–cell junctions. Interstitial flow arises from fluid movement within the tissue surrounding the ECs, shearing ECs on their basal side. For large vessels, interstitial flow can originate from transmural flow currents as well as from other sources such as fluid leakage from the vaso vasora. In the microvasculature, interstitial flow stems from porous medium flow in the surrounding parenchyma.

In vitro, physiological levels of transmural flow increase endothelial sprouting^169^ both in the cases of outward^170,171^ and inward flow^172,173^, with more filopodial protrusions under inward flow^169^. Transmural flow is also necessary for sustained sprout elongation^170^. In a microfluidic branching model, transmural flow was shown to restore sprouting after inhibition by luminal shear stress^174^. Interestingly, the shear stress threshold for triggering angiogenesis is conserved between luminal and transmural flow, at ~1 Pa^170^.

Because interstitial flow can have multiple sources, its intensity is highly variable across the vasculature and is difficult to measure in vivo, with the few reported velocities varying between 0.1 and 4 μm/sec^168,175^. In vitro, interstitial flow around ECs embedded within a 3D matrix stimulates network formation^176–178^. Vascular tubes align in the flow direction^179^, with vascular sprouts elongating against the flow direction^176,180^.

#### Influence of EC type and cell density

Arterial and venous ECs exhibit different responses to flow-induced shear stress. For instance, arterial ECs become more polarized^181^ and exhibit more prominent actin stress fibers in the direction of flow than venous ECs^131,182,183^. It is also notable that certain types of ECs such as aortic valve ECs or lymphatic ECs behave differently with alignment orthogonal to the direction of flow^184,185^. Similarly, brain microvascular ECs subjected to a steady shear stress of 1.6 Pa do not elongate or align and continue to exhibit a randomly oriented cytoskeleton^186,187^.

Although EC responses to flow have been investigated at both the single cell and monolayer levels, very few studies have specifically tackled the influence of cell density on EC flow responses. One study reported that only confluent ECs aligned in response to a 2 Pa shear stress^188^. Cell density also influences EC migration: while low density ECs migrate in the direction of the flow, ECs in dense monolayers move against the flow direction^136^.

## Compressive stresses from blood pressure

Blood pressure exerts a normal force that compresses the EC apical surface. Blood pressure varies drastically along the vasculature, from ~1.3 kPa (10 mmHg) in veins up to ~16 kPa (120 mmHg) in the human aorta during systole. Severe hypertension may lead to pressures as high as ~27 kPa (200 mmHg). Blood pressure is highly pulsatile on the arterial side, but these oscillations are progressively dampened by the vessel elasticity until they virtually disappear in capillaries.

Hydrostatic pressure applied to ECs in vitro influences cell shape, cytoskeletal organization, and various aspects of vascular function. Bovine aortic ECs (BAECs) under both physiological (5–20 kPa) and low pressure (0.1–1 kPa)^189,190^ elongate while maintaining a random orientation. They also have a smaller area and less smooth cell contours^191–194^. However, some studies applying similar pressures reported no elongation in BAECs^163,195,196^. Interestingly, HUVECs under physiological pressure also have a reduced cell area but do not exhibit the elongation and tortuosity responses^197,198^, suggesting that pressure-induced changes in cell shape are specific to arterial ECs. The morphological changes are mirrored by cytoskeletal reorganization, with the formation of central stress fibers and remodeling of FAs under both physiological^163,192,194^ and low pressure values^189,190,199^. Pressure-induced cytoskeletal reorganization follows a two-step dynamic: increased cytoskeletal tension through actomyosin-mediated contraction after 1 h followed by increased cortical actin density through actin polymerization and assembly of stress fibers^198,200^.

Hydrostatic pressure at both physiological^197^ and sub-physiological levels^199^ increases EC proliferation, consistent with reports of increased angiogenesis and tubulogenesis in these situations^198,201^. For low and physiological ranges of pressure, the rate of proliferation correlates with the magnitude of the applied pressure^189,193^, while pathological pressures (20–25 kPa) induce EC degeneration and apoptosis^202,203^.

One hypothesis put forward to explain pressure-induced EC proliferation is the disruption of adherens junctions^192,204^, leading to a multilayer EC structure in vitro^189–192^. Indeed, the formation of VE-cadherin-based junctions normally inhibits proliferation within confluent monolayers^205,206^. The multilayer structure is not observed in subconfluent ECs under pressure^193^, which confirms that it originates from an excessive proliferation of confluent ECs. Physiological pressure further alters endothelial function by increasing intercellular gaps^197^, inducing a reversible loss of monolayer integrity^191,207^, loss of barrier function^200^, and increased permeability^204^. In contrast, low pressure was shown to protect pulmonary endothelial monolayer integrity against inflammatory agents^208^.

Unlike constant pressure, the effect of pulsatile pressure on ECs in vitro has received little attention. Similar to constant pressure, a magnitude-dependent effect of pulsatile pressure on proliferation was reported, with physiological pressure values increasing proliferation and pathological values decreasing it^196,201^. Application of pulsatile pressure also leads to a magnitude-dependent decrease in peripheral actin and relocalization of ZO-1 junctions associated with decreased permeability^209^.

### Tensile stresses

Tensile stresses in the vasculature can be axial, due to tissue growth or movement, or circumferential (hoop), due to the transmural pressure difference which dilates the vessels cyclically. In vivo measurements report a wide range of axial strain magnitudes across the vasculature: ~20% in the lungs^210^, 5% in coronary arteries^211^, and 5–15% in leg arteries^212–214^. Hoop strains due to diameter changes during the cardiac cycle are in the range of 0–15%^213,215–218^. In most in vitro studies, 5–10% strains are considered physiological while 15–20% strains are viewed as pathological.

#### General cell response: alignment, elongation, and activation

In vitro studies that have examined the effect of stretch on ECs all report EC elongation and alignment orthogonal to the strain direction^219–221^. This result is in line with the longitudinal alignment of ECs in vivo, orthogonal to the direction of circumferential strain. This behavior is highly dynamic: when the direction of the strain is changed during the experiment, ECs reorient orthogonal to the new strain direction^220^. The cell morphological changes are reflected intracellularly by orthogonal alignment of actin filaments and an increased number of stress fibers^222,223^. At the onset of stretch, stress fibers disassemble^224,225^ with vanishing traction forces^226^, then stress fibers reassemble in the transverse direction within minutes^222,224,227,228^ with recovery of the transverse traction forces, and finally the entire cell reorients within hours^221,226^. Stretching the substrate upon which cells adhere increases tension in the actin cytoskeleton^229^, increasing cell stiffness^230,231^.

Another major effect of strain is EC activation: low strains (5–10%) inhibit apoptosis and increase proliferation, while large strains (15–20%) have the opposite effect^232–235^. The stretch-induced proliferation requires cell–cell junctions^236–239^. An intermediate level of strain (10%) is also able to increase endothelial motility and migration^240,241^, tubulogenesis, and endothelial sprouting^235,242^ and aligns the newly formed sprouts orthogonal to the strain^243,244^. The stimulation of angiogenesis is observed for both static and cyclic tensile strains.

#### Stretch direction: free uniaxial, pure uniaxial, and biaxial

**Fig. 4:**
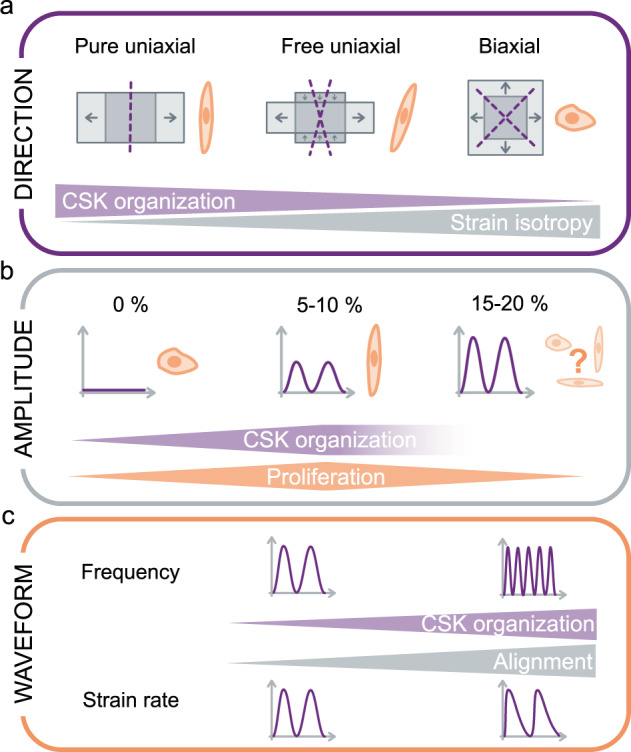
Features of tensile stresses experienced by endothelial cells. **a** Strain direction can be purely uniaxial, free uniaxial (with an additional lateral compression), or biaxial (isotropic). Purple dotted lines indicate minimum strain direction. Strain direction influences cytoskeletal (CSK) organization. **b** Strain amplitude influences cytoskeletal organization and cell proliferation, which are maximized for intermediate strain amplitudes. **c** Strain frequency and strain rate influence cytoskeletal organization and cell alignment.

Beyond the general observation of EC alignment orthogonal to strain, variations in the exact orientation angle have been reported. These apparent discrepancies are reconciled when viewed through the lens that ECs align in the direction of minimal strain^221,223^. Three major forms of stretching can be applied to cells: free uniaxial, pure uniaxial, and biaxial (Fig. 4a). In free uniaxial stretching, the substrate on which the cells are cultured is elongated actively along one axis but undergoes slight retraction in the orthogonal direction due to the positive Poisson’s ratio exhibited by most materials. In pure uniaxial stretching, the sample is constrained orthogonal to the uniaxial stretch in order to prevent the orthogonal (transverse) retraction strain. In biaxial stretching, the sample is stretched in two orthogonal directions via two unidirectional stretches of a rectangular sample, radial stretching of a circular sample, or by inflation of a circular membrane.

The first experiments with pure uniaxial stretching were conducted in the late 1990s^221^, with a follow-up study that specifically explored the variation of cell orientation among the three types of stretchers^223^. Cells were found to orient in the minimal strain direction (90° for pure uniaxial stretching^220,223,245^ and ~70° for free uniaxial stretching^223,246^), which is determined by the balance between the longitudinal tensile strain and the transverse compressive strain^228^. ECs were also found to avoid pure compressive strains, albeit with slower dynamics^247^.

In the biaxial stretch configuration, cells are subjected to two different minimum strain directions, leading to a cuboidal morphology^223,245,248^. These platforms are mostly used to investigate functional responses to stretch, such as cell proliferation or changes in gene expression. Inflation of circular membranes generates equiaxial strain at the center, where cells are seen to be randomly oriented, and a gradient of anisotropic biaxial strain in the peripheral areas which complicates interpretation of cell orientation responses^249,250^. In contrast, radial stretching of circular membranes leads to uniform equiaxial strains^248^.

#### Stretch pattern: amplitude, frequency, and waveform

EC response to cyclic stretch loading is modulated by the stretch pattern, most notably its frequency, amplitude, and precise waveform (Fig. 4b, c). Increasing the frequency towards the physiological value of 1 Hz amplifies cell response, leading to more elongated cells with an orientation angle closer to the minimum strain direction^251^ and with a faster response^252^. A minimum frequency of 0.1 Hz for cyclic strain is necessary to induce a response^251,253,254^. Similarly, the stretch amplitude has a strong influence on EC mechano-response. Large physiological strains (~10%), induce a final orientation that is closer to the “minimum strain direction”^228,247,255^, reduced orientation dispersion^220^, and accelerated dynamics^252^ relative to smaller strains (~5%). For pathological strains, above 15%, the results are less clear, with reports of both orthogonal^252,256^ and longitudinal alignment^250^. Possible explanations are differences in cell type (venous vs. arterial ECs) or in stretching profiles (free uniaxial vs. biaxial). Finally, physiological waveforms, i.e. faster extension and slower relaxation, lead to more intense responses with faster dynamics^252,257^, as ECs are more responsive to tensile strain rate than to compressive strain rate^258^.

#### Influence of EC type and cell density

Single cells align in the direction of a 2% compressive strain while confluent monolayers align in the direction of the true minimum strain^259^. Cell confluency also modulates the response to strain amplitudes, suggesting an important role for cell–cell junctions^259^. The previously described switch of endothelial orientation from orthogonal to parallel to the stretch direction under pathological strain levels is lost when cell–cell junction formation is inhibited via blocking antibodies^250^. This collective orientation switch is hypothesized to be key for preserving monolayer integrity, which can be damaged under pathological strains^260^.

An additional layer of complexity is mechanical preconditioning. EC confluent monolayers grown under cyclic strain withstand deformation while their static counterparts are less compliant and detach from the substrate under strain^224^. Interestingly, the denudation zones correlate with areas of initial higher density, which is known to modulate cytoskeletal tension and substrate adhesion^205^, two key components of the strain sensing mechanism. Lastly, as strain levels vary along the vascular tree, EC type (arterial, capillary, or venous) is likely to modulate its mechano-response. One study tackled this question and found that only venous ECs reorient while only arterial ECs exhibit increased proliferation in response to stretch^222^. The dependence of the stretch response on EC type merits further investigation in light of the fact that most of the other studies reviewed in this section have reported orthogonal alignment despite using arterial ECs, and a number of studies using venous ECs have reported increased proliferation^235,236,244^.

## Combined cues

While the study of individual substrate- and flow-derived mechanical cues on ECs has greatly improved our understanding of the fundamental mechanobiology of these cells, ECs in vivo experience a complex combination of all these cues. The recent development of experimental platforms integrating multiple mechanical stimuli has begun to address this issue. Depending on their respective magnitudes, waveforms, directionality, and the cellular processes they regulate, multiple cues may have synergistic or antagonistic effects when they regulate the same cellular process(es), or one cue may modulate the effect of another on a cellular response that it does not directly regulate on its own.

### Flow and substrate cues

#### Flow and topography

How apical flow-derived shear stress and basal contact stresses due to BM topography cooperate or compete to modulate EC responses is a central question, for optimizing the design and surface properties of endovascular devices. When flow and anisotropic topographies in the form of ridges/grooves are oriented in the same direction, synergistic effects on ECs are observed. For instance, EC orientation and elongation are more pronounced for the combination of both cues than for either one alone^137,261^. ECs on grooves under flow also exhibit increased adhesion forces^51,262^, leading to higher resistance to detachment^41^ and a protective effect on monolayer integrity^51,137^. They also show enhanced counterflow migration^263,264^ and thus more efficient wound healing^263^.

When the flow and anisotropic topography cues are oriented orthogonal to one another, most studies report that ECs align and elongate in the direction of the topography rather than the flow^261,265^. However, this conclusion depends on both the groove dimensions, with micron-scale grooves counteracting the effect of flow more effectively than nanoscale grooves^261^, and the magnitude of shear stress, with ECs aligning perpendicular to the grooves for sufficiently high shear stress levels^266^.

In vivo, ECs that are elongated and aligned in the direction of blood flow exhibit an anti-inflammatory, atheroprotective phenotype whereas ECs in disturbed flow zones are largely cuboidal and have a pro-inflammatory and atheroprone profile^267^. An interesting question is whether the differences in inflammation phenotype are driven directly by the differences in cell morphology which can also be modulated by the underlying topography. Studies on ECs on grooved substrates in the absence of flow^30,39,268^ suggest that cell morphology regulates phenotype independently of flow. Nevertheless, a cooperative effect of the two types of cues has also been reported, with aligned collagen fibers reducing EC inflammation under disturbed flow^265^.

#### Flow and substrate stiffness

How vascular wall stiffening with age or with vascular disease affects EC responses to blood flow has motivated studies of the combination of different substrate stiffnesses with flow-induced shear stress. As an isotropic biophysical cue, substrate stiffness appears to modulate other directional cues. For instance, higher shear stresses (2.2 vs. 0.6 Pa) are required to align ECs on very soft substrates (elastic modulus of 100 Pa) compared to stiffer substrates (10 kPa)^269^. Kohn et al. nicely demonstrated that stiffness values characteristic of healthy vessel walls (2.5 kPa) promote the atheroprotective signals induced by fluid shear stress compared to stiffer substrates^270^. Thus, it appears that both exceedingly soft and pathologically stiff substrates decrease EC sensitivity to flow.

### Stretch and substrate cues

The interaction between stretch and substrate cues has mostly been investigated on cell types other than ECs such as fibroblasts or cells derived from mesenchymal stem cells. As these studies reveal interesting combined effects, we review the broad findings that should guide future studies on ECs.

#### Stretch and substrate topography

For adhesion-based substrate cues, cell elongation and orientation are driven by the pattern anisotropy, despite the competing strain^271–273^. When strain competes with anisotropic substrate topography cues, the dominant cue depends on the characteristic size of the topography: for microgrooves, cells align with the topography^274,275^, whereas for nanogrooves^276^ or cell-scale micropillars^277^, the strain dictates cellular orientation. Interestingly, it appears that while the basal actin and cell shape follow the contact guidance of elliptical micropillars, the apical actin and nucleus orientation are dictated by the strain^278^, underscoring the complexity of the competition between topography and stretch. When topography and stretch are configured to reinforce one another, they have a synergistic effect as illustrated by the amplification of fibroblast alignment on grooved substrates in the presence of orthogonal stretch^279^.

#### Stretch and substrate stiffness

Substrate stiffness alters cellular responses to stretch, with soft substrates either attenuating cell alignment^280^ or even changing the alignment direction from orthogonal to parallel to the stretch direction^281^. Conversely, stretch rescues the impaired cell spreading observed on soft substrates^280,282^. Both substrate stiffness and stretch modify cytoskeletal tension, suggesting the following possible mechanism to explain these results: stretch restores cytoskeletal tension, allowing cell spreading on soft substrates, and soft substrates decrease cytoskeletal tension allowing cells to withstand the additional tension due to alignment parallel to stretch. One study cultured lung ECs on a stretchable membrane of physiological stiffness to mimic the native soft lung matrix and respiration-induced strains^283^. In this system, a synergistic effect of stiffness and stretch was observed, protecting the cells against inflammatory thrombin.

Finally, stretch and substrate stiffness are tightly coupled, with stiffness determining the level of strain for a given stress and substrate stiffness being affected by stretch. In fibrous matrices (such as collagen) that exhibit strain-stiffening behavior^284^, uniaxial stretching leads to anisotropic stiffening which aligns cells in the stiffer direction, i.e., the strain direction^281,285^. Interestingly, cells already aligned by anisotropic stiffness exhibit enhanced alignment in response to cyclic stretch, indicative of a synergistic effect of stiffness anisotropy and stretch^281^.

### Shear stress and stretch

Shear stress and circumferential stretch both arise from blood hemodynamics and are therefore naturally coupled both in vivo and in vitro. As already described, ECs align parallel to shear stress and perpendicular to stretch; thus, axial shear stress and circumferential strain are expected to reinforce one another while shear stress and axial stretch would be expected to counteract one another. ECs subjected to strain orthogonal to an applied shear stress (either steady or pulsatile) indeed exhibit more pronounced^286,287^ and faster^288^ alignment as well as more prominent actin stress fibers^289^. In contrast, stretch parallel to shear stress leads to competition between the two stimuli, with the aggregate effect depending on the relative magnitudes of the cues. More specifically, a shear stress of 0.5 Pa dominates the effect of strain (even for strains of 15%), whereas a shear stress of 0.08 Pa is dominated by the strain^245^. Owatverot et al. introduced the notion of “equipotent stimuli”: the levels of stresses needed to elicit similar responses in terms of amplitude and dynamics^288^. They showed that equipotent shear and cyclic stretch cancel one another when applied in counteracting configurations. Interestingly, if the angle between the shear stress and the strain is intermediate (30°–60°), both stimuli influence the response^245^.

In vivo, pulsatile shear stress and cyclic hoop stretch are not synchronized, exhibiting a phase shift whose magnitude varies across the vascular tree. This phase shift was demonstrated to attenuate the synergistic effect, with an altered production of vasodilators^290^ and an increased expression of atherogenic genes when both stimuli are perfectly out of phase^291^.

## Conclusions and future directions

In this review, we have described how different forms of mechanical stimulation regulate vascular EC structure and function. Engineered in vitro systems have shed light onto fundamental EC responses to individual mechanical cues, such as cell alignment in the direction of either an applied shear stress or an anisotropic substrate topography and orthogonal to an applied strain. We have also touched upon the need to better characterize possible mechanobiological differences among ECs derived from different vascular beds as well as between single cells and cellular monolayers.

To design and implement systems and experiments that address physiologically relevant questions in mechanobiology, it is essential to carefully characterize the detailed nature of the forces at play. This includes the direction of the forces (isotropic or anisotropic for topography, axial or radial for flow or strain), the dimension/magnitude of the forces (scale of topography, values of strain, shear stress, or stiffness), and, when applicable, the time-dependent pattern of the force (waveform and frequency of strain or flow profile, for instance). Much work is needed to better understand the effect of multiple mechanical cues on ECs and to elucidate the mechanisms by which ECs integrate and decipher multiple environmental signals, as discussed in the last section. In this regard, the notion of “equipotent stimuli” provides an attractive framework for translating the effects of different types of cues into a common “language” that the cell uses to integrate multiple mechanical cues exerted simultaneously. Applying this framework to both physiological and pathological conditions would define the normal “equilibrium” values of the individual stimuli and how pathologies that alter one or more of the biophysical cues would perturb this equilibrium. Synergistic effects are also reported for almost all combinations of cues, which highlights the robustness of the vascular system, as well as potential compensatory mechanisms to maintain homeostasis to the extent possible in pathological settings.

It should be noted that the different mechanical cues are often interdependent, underscoring the need for careful decoupling of their respective contributions. For instance, the topography of the substrate and the waviness of the EC surface modify the shear stress that the cells perceive^292–294^. Similarly, substrate stiffness changes the effective strain applied on the cells^281^. In a pathological context, BM thickening (up to twofold) in pathologies such as diabetes^295,296^ or atherosclerosis^297,298^ is expected to change both the topography and stiffness sensed by ECs and consequently the strain that ECs undergo. All of these aspects should therefore be carefully taken into account in the design of experimental systems and in the interpretation of the obtained results.

Cellular mechanobiology is an active field of research that nicely complements the more traditional biochemically-centered view of most biological processes. As we have highlighted throughout this review, mechanical forces are particularly diverse, dynamic, and multifaceted in the vascular system, and these forces play a critical role in regulating vascular physiology and pathology. In light of the fact that many of these forces are borne most directly by the endothelium, elucidating the mechanisms governing EC mechanobiology will enhance our understanding of the etiology of various vascular diseases including atherosclerosis, thrombosis, aneurysm formation, and diabetes. Furthermore, understanding how substrate- and flow-derived stresses regulate EC structure and function promises to inform the design and development of next generation implantable endovascular devices including stents, valves, and grafts.

One of the principal challenges in mechanobiology in the coming years is certainly technological. Two recent developments provide unique opportunities for devising novel in vitro systems that promise to greatly enhance our understanding of endothelial mechanobiology. The first development is the democratization of previously complex and expensive techniques, leading to better integration of mechanical cues. An example is the field of microfabrication where techniques such as micropatterning and microfluidics have become much more widely available in the past decade. Another example is the advent of DIY technologies such as Lego-based stretchers^299^ or paper-based compression-flow devices^300^. As a result, mechanical platforms can be more readily adapted to investigate particular diseases, as illustrated by a recent study modeling atherosclerosis on a chip^60^. The second development entails technological advances that allow the fabrication of innovative in vitro platforms, thus enabling new combinations of mechanical cues^301^. For instance, the topographical patterning of hydrogels^302–304^, or hydrogel laser carving, recently enabled the production of perfusable endothelialized capillaries inside a soft hydrogel^305^. Future systems promise to better recapitulate the complexity of the native vascular environment and to provide finer control over the detailed magnitudes, directions, and waveforms of the applied biophysical stimuli, thereby furthering our understanding of EC responses to mechanical forces.
